# Nonstationary Stochastic Responses of Transmission Tower-Line System with Viscoelastic Material Dampers Under Seismic Excitations

**DOI:** 10.3390/ma18051138

**Published:** 2025-03-03

**Authors:** Mingjing Chang, Bo Chen, Xiang Xiao, Yanzhou Chen

**Affiliations:** 1 School of Civil Engineering and Architecture, Wuhan University of Technology, Wuhan 430070, China; changmj@whut.edu.cn; 2 School of Transportation and Logistics Engineering, Wuhan University of Technology, Wuhan 430070, China; xiangxiao@whut.edu.cn; 3 Central-South Architectural Design Institute Co., Ltd., Wuhan 430071, China; dly123home@163.com

**Keywords:** TTL system, stochastic seismic response, VMD, control performance, nonstationary

## Abstract

The excessive vibration or collapse of a transmission tower-line (TTL) system under seismic excitation can result in significant losses. Viscoelastic material dampers (VMDs) have been recognized as an effective method for structural vibration mitigation. Most existing studies have focused solely on the dynamic analysis of TTL systems with control devices under deterministic seismic excitations. Studies focusing on the nonstationary stochastic control of TTL systems with VMDs have not been reported. To this end, this study proposes a comprehensive analytical framework for the nonstationary stochastic responses of TTL systems with VMDs under stochastic seismic excitations. The analytical model of the TTL system is formulated using the Lagrange equation. The six-parameter model of VMDs and the vibration control method are established. Following this, the pseudo-excitation method (PEM) is applied to compute the stochastic response of the controlled TTL system under nonstationary seismic excitations, and a probabilistic framework for analyzing extreme value responses is developed. A real TTL system in China is selected to verify the validity of the proposed method. The accuracy of the proposed framework is validated based on the Monte Carlo method (MCM). A detailed parametric investigation is conducted to determine the optimal damper installation scheme and examine the effects of the service temperature and site type on stochastic seismic responses. VMDs can effectively suppress the structural dynamic responses, with particularly stable control over displacement. The temperature and site type have a notable influence on the stochastic seismic responses of the TTL system. The research findings provide important references for improving the seismic performance of VMDs in TTL systems.

## 1. Introduction

Earthquakes may lead to the excessive vibration and plastic failure of high-rise tower structures, resulting in structural destruction and even failure, causing significant economic losses [1,2]. Traditional methods for suppressing structural vibrations are often uneconomical because they focus on increasing stiffness to enhance its load-bearing capacity. An alternative approach to protect engineering structures is the application of various control devices [3,4]. To ensure the normal operation of transmission tower-line (TTL) systems, the application of energy dissipation devices in transmission towers is widely recognized as an effective control measure [5,6]. These energy dissipation devices can absorb and dissipate vibration energy and mitigate the dynamic impact induced by external excitations, thereby reducing the vibration amplitude of the structures, minimizing damage risk, and, ultimately, ensuring the safe operation of TTL systems [7,8,9]. Semi-active control devices, such as magnetorheological dampers, can effectively reduce the dynamic responses of high-rise towers. However, their configuration and control systems are commonly complicated, and the expense is relatively high. Therefore, the real application of semi-active devices in the vibration control of large truss towers has rarely been reported. To this end, various passive control devices, such as viscoelastic material dampers (VMDs), friction dampers, electromagnetic inertial mass dampers, etc., have been used in the vibration control of high-rise truss towers [10,11]. VMDs, characterized by their simple structure, easy installation, and excellent energy dissipation performance [12,13], have been widely applied in various fields, such as architecture, aerospace, and mechanical engineering, for vibration mitigation [14,15,16]. VMDs can suppress the vibration responses of TTL systems by increasing the overall structural damping ratio [17]. Zeng et al. [18] proposed a vibration mitigation calculation method for transmission towers with additional VMDs to determine the equivalent damping ratio. Huang et al. [19] studied the optimization design of VMDs for the response control of transmission towers.

It is noted that the majority of existing studies have focused solely on the dynamic analysis of TTL systems under deterministic seismic excitations. Seismic motions are inherently stochastic excitations with pronounced nonstationary characteristics. The seismic process, spanning from initiation to termination, is generally classified as a nonstationary stochastic phenomenon. In the seismic response control of TTL systems, the stochastic vibration analysis method based on power spectral density (PSD) is commonly taken as a robust and versatile approach. According to classical stationary stochastic vibration theory, Priestley [20] advanced the theoretical framework for assessing nonstationary stochastic responses. Most extensive research has been conducted on nonstationary stochastic seismic response analysis for high-rise and bridge structures [21,22,23]. Studies focusing on the nonstationary stochastic control of TTL systems with VMDs have not been reported. Zhong et al. [24] developed formulas to compute the background and resonant responses of transmission towers subjected to nonstationary downbursts by a nonstationary modal analytical method. Zhang et al. [25] developed an analytical approach to evaluate the extreme non-stationary dynamic responses of transmission towers under downbursts in the frequency domain. However, their computation focused on a single transmission tower, neglecting the coupling effects of tower-line systems.

The installation of VMDs may introduce damping non-orthogonality in a TTL system, and, thereby, the modal decomposition methods are unsuitable for solving the vibration equations. Therefore, the precise analysis of the nonstationary stochastic seismic responses of a TTL system with control devices is quite different. In this regard, a comprehensive analytical framework for the nonstationary stochastic responses of TTL systems with VMDs under stochastic seismic excitations is proposed in this study. Initially, the analytical model of the TTL system is formulated using the Lagrange equation. A six-parameter model of VMDs is introduced, and a control method based on VMDs for TTL systems is proposed. Following this, the pseudo-excitation method (PEM) is applied to compute the stochastic response of the controlled TTL system under nonstationary seismic excitations, and a probabilistic framework for analyzing extreme value responses is developed. Four damper schemes are taken into account to determine a rational VMD location. A detailed parametric investigation is conducted to investigate the effects of the service temperature and site type on stochastic seismic responses.

## 2. Model of TTL-VMD System

### 2.1. Mechanical Model of TTL

**Figure 1:**
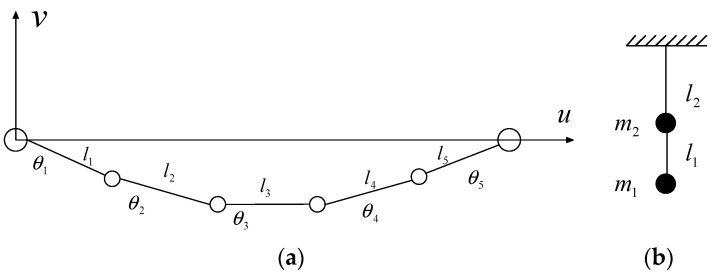
Model of a transmission line for different horizontal directions: (**a**) in-plane vibration; (**b**) out-of-plane vibration.

The nonstationary stochastic dynamic computation of transmission lines requires numerous iterative calculations and is time-consuming. Therefore, the Hamilton principles and the Lagrange equation are used to develop the analytical model of transmission lines [26,27,28,29]. As displayed in Figure 1, a transmission line can be modeled as a cable by several lumped masses and elastic elements. Following the Hamilton principles, the generalized coordinates of the line can be selected as the element angle *θ* and the length *l*. In the in-plane direction, the kinetic energy and potential energy of the line can be determined by the Lagrange formulation. The structural equation of motion is derived using generalized coordinates:

(1) $$\int \delta \left[T_{c}(t)-U_{c}(t)\right]dt+\int \delta W_{c}(t)dt=0$$

in which *W_c_*(*t*) is the virtual work of the line; *T_c_* and *U_c_* are the kinetic energy and potential energy of the line.

The mass matrix $M_{l}^{in}$ and stiffness matrix $K_{l}^{in}$ of the line are determined by computing the partial differential of kinetic energy and potential energy to the generalized velocity and displacement. In the out-of-plane direction, the line can be simplified as a pendulum, and the structural system matrices are expressed as follows:

(2) $$M_{l}^{out}=\left[\begin{array}{cc} m_{1} & \\ & m_{2} \end{array}\right]$$

(3) $$K_{l}^{out}=\left[\begin{array}{cc} \frac{m_{1}g}{l_{1}} & -\frac{m_{1}g}{l_{1}}\\ -\frac{m_{1}g}{l_{1}} & \frac{m_{1}g}{l_{1}}+\frac{(m_{1}+m_{2})g}{l_{2}} \end{array}\right]$$

where $M_{l}^{out}$ and $K_{l}^{out}$ are the structural mass and stiffness matrices, respectively.

**Figure 2:**
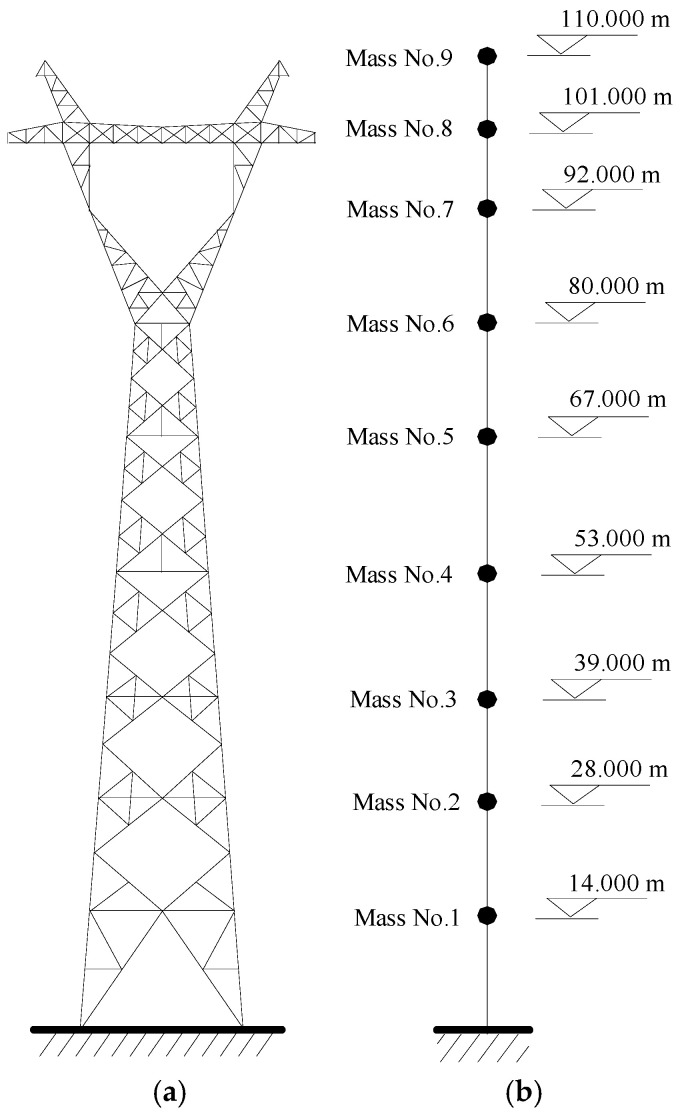
Mechanical model of the tower: (**a**) 3D finite element model; (**b**) lumped model.

**Figure 3:**
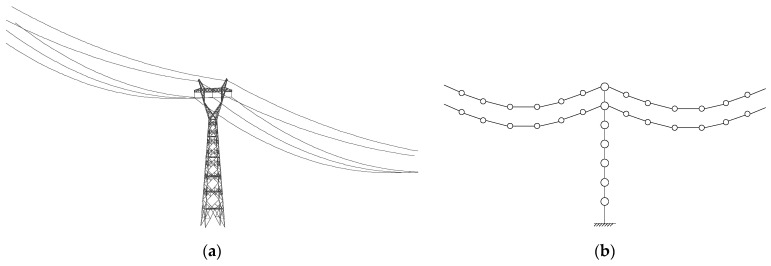
Analytical model of a TTL system: (**a**) 3D finite element model; (**b**) lumped model.

As displayed in Figure 2a, a transmission tower is a typical high-rise flexible structure, and the analytical model can be constructed using the software ABAQUS (version 6.14). If a three-dimensional (3D) finite element model is used for real transmission towers with control devices, the nonstationary stochastic dynamic computation will be complicated and time-consuming. In this regard, lumped-mass models (Figure 2b) are commonly adopted in the stochastic dynamic analysis and vibration control of transmission towers [6]. The analytical model of the TTL is constructed as shown in Figure 3.

### 2.2. Mechanical Model of VMDs

Viscoelastic materials can be used in civil engineering structures to dissipate vibration energy in wind- and earthquake-induced vibration. Many factors, such as ambient temperature, frequency, and strain, may affect the behavior of viscoelastic materials. The shear stress τ(t) is given by

(4) $$\tau (t)=G^{\prime }(\omega )\gamma (t)+\frac{G^{\prime \prime }(\omega )}{\omega }\dot{\gamma }(t)$$

in which γ(t) is the shear strain that varies with time *t*; *ω* is the frequency of the harmonic shear strain; $G^{\prime }(\omega )$ is the shear storage modulus; and $G^{\prime \prime }(\omega )$ is the shear loss modulus.

**Figure 4:**
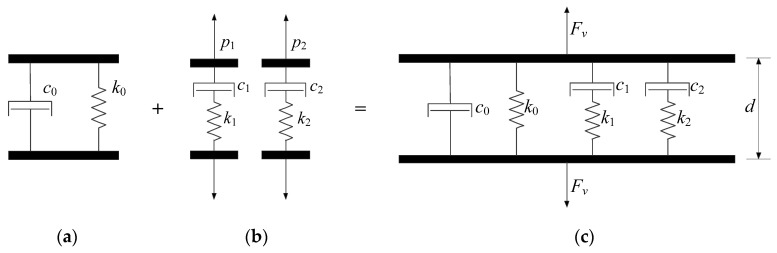
Six-parameter model of VMD: (**a**) Kelvin element; (**b**) two Maxwell elements; (**c**) six-parameter model.

To describe the frequency-dependent behavior of VMDs, many studies have employed the combination of multiple elastic springs and dashpots, such as the generalized Kelvin and generalized Maxwell models [30,31,32,33]. However, these models are relatively complex, and theoretical analysis typically requires simplicity. In this study, the six-parameter model proposed by Mazza and Vulcano is used to describe the VMD model, which can be regarded as a parallel combination of two Maxwell elements and one Kelvin element [34,35], as shown in Figure 4. The Kelvin element is composed of a dashpot with a damping coefficient *c*_0_ and a spring with a stiffness coefficient *k*_0_ in parallel. Each Maxwell element is composed of a dashpot with damping coefficient *c*_1_ (or *c*_2_) and a spring with stiffness coefficient *k*_1_ (or *k*_2_) connected in series.

The storage stiffnesses *K*′ and loss stiffness *K*″ can be expressed, for a given frequency *ω*, as functions of the constants characterizing the behavior of the springs and dashpots constituting the six-parameter model:

(5) $$K^{\prime }\left(\omega \right)=\omega^{2}\left(\frac{k_{1}c_{1}^{2}}{\omega^{2}c_{1}^{2}+k_{1}^{2}}+\frac{k_{2}c_{2}^{2}}{\omega^{2}c_{2}^{2}+k_{2}^{2}}\right)+k_{0}$$

(6) $$K^{\prime \prime }\left(\omega \right)=\omega \left(\frac{k_{1}^{2}c_{1}}{\omega^{2}c_{1}^{2}+k_{1}^{2}}+\frac{k_{2}^{2}c_{2}}{\omega^{2}c_{2}^{2}+k_{2}^{2}}+c_{0}\right)$$

If Equations (5) and (6) are divided by the shape coefficient (*A*/*h*, where *A* denotes the total shear area, and *h* denotes the total thickness), analogous equations can be written expressing the storage modulus $G^{\prime }(\omega )$ and the loss modulus $G^{\prime \prime }(\omega )$ as functions of the constants of the six elements.

Thus, the force–displacement relationship of the VMD is given by

(7) $$F_{v}=k_{0}d+c_{0}\dot{d}+p_{1}+p_{2}$$

where *F_v_* denotes the force at both ends; *d* and $\dot{d}$ denote the displacement and velocity of the damper, respectively; *p*_1_ and *p*_2_ denote the damper forces of the Maxwell elements, respectively. The relationships between the damper forces and displacement can be expressed as

(8) $$\left\{\begin{array}{c} {\dot{p}}_{1}+\mu_{1}p_{1}=k_{1}\dot{d}\\ {\dot{p}}_{2}+\mu_{2}p_{2}=k_{2}\dot{d} \end{array}\right.$$

where *μ*_1_ and *μ*_2_ are the reciprocal of the relaxation time of the Maxwell elements, satisfying the conditions *μ*_1_ = *k*_1_/*c*_1_ and *μ*_2_ = *k*_2_/*c*_2_, respectively.

**Figure 5:**
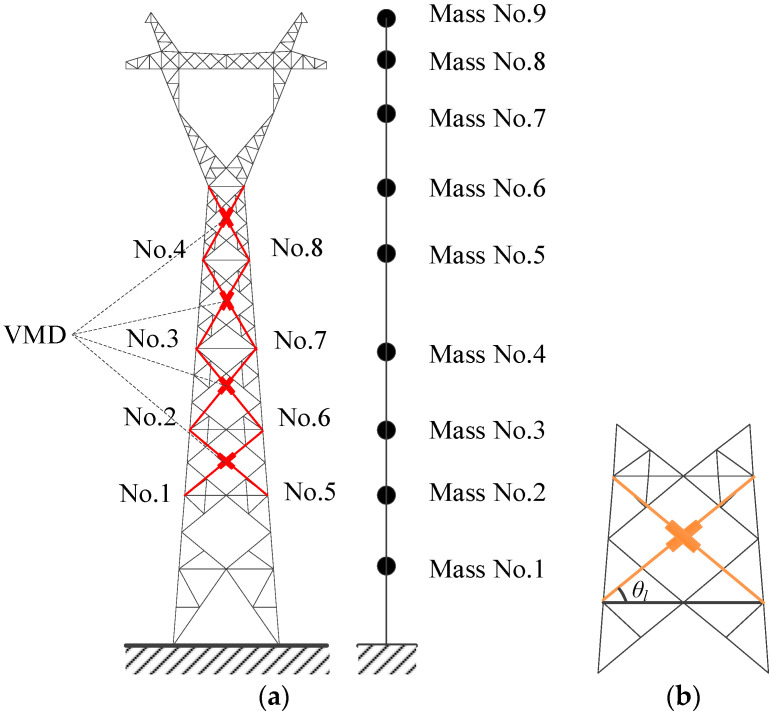
Damper installation scheme of the TTL system: (**a**) location of VMDs; (**b**) damper installation scheme.

The damper is installed diagonally between the adjacent nodal layers in the transmission tower, as shown in Figure 5. The angle *θ_l_* represents the angle between the damper installed in the *l*th layer (*l* = 1, 2, …, *n*; *n* is the number of VMDs) and the corresponding layer, as shown in Figure 5b. Since the damper force is generated by the relative displacement between the two end nodes of the damper, the damper force *F_vl_* in the *l*th layer can be expressed as

(9) $$F_{vl}=k_{0l}d_{l}\cos\theta_{l}+c_{0l}{\dot{d}}_{l}\cos\theta_{l}+p_{1l}+p_{2l}$$

where *c*_0*l*_ and *k*_0*l*_ are the damping coefficient and stiffness coefficient of the Kelvin element for the damper in the *l*th layer, respectively; *d_l_* is the relative displacement between the *l*th layer and the (*l* − 1)th layer of the transmission tower, satisfying the condition *d_l_ = y_l_* − *y_l−_*_1_; *y_l_* denotes the displacement of the *l*th layer; and *θ_l_* is the angle between the damper and the *l*th layer. The damper forces of a Maxwell element are as follows:

(10) $$\left\{\begin{array}{c} {\dot{p}}_{1l}+\mu_{1l}p_{1l}=k_{1l}{\dot{d}}_{l}\cos\theta_{l}\\ {\dot{p}}_{2l}+\mu_{2l}p_{2l}=k_{2l}{\dot{d}}_{l}\cos\theta_{l} \end{array}\right.$$

where *μ*_1*l*_ and *μ*_1*l*_ are the reciprocal of the relaxation time of the Maxwell elements in the *l*th layer, satisfying the conditions *μ*_1*l*_ = *k*_1*l*_/*c*_1*l*_ and *μ*_2*l*_ = *k*_2*l*_/*c*_2*l*_, respectively; *k*_1*l*_ and *k*_2*l*_ denote the stiffness coefficients of the Maxwell elements in the *l*th layer; and *c*_1*l*_ and *c*_2*l*_ denote the damping coefficients of the Maxwell elements in the *l*th layer.

## 3. Equation of Motion of TTL System with VMDs

To be an energy-dissipating device, VMDs can be utilized in engineering structures for vibration mitigation. The equation of motion of the controlled TTL system with VMDs under nonstationary seismic excitation is given by

(11) $$M\ddot{y}(t)+C\dot{y}(t)+Ky(t)+F_{v}(t)=MI{\ddot{x}}_{g}(t)$$

where y(t), $\dot{y}(t)$, and $\ddot{y}(t)$ are the displacement, velocity, and acceleration responses of the controlled TTL system with VMDs, respectively; $F_{v}(t)$ is the damper force vector of VMDs; M, C, and K are the mass, damping, and stiffness matrices of the controlled system with VMDs, respectively; **I** is the identity matrix; and ${\ddot{x}}_{g}(t)$ is the nonstationary seismic acceleration excitation, including both intensity and frequency non-stationarities.

The system matrices and vectors with VMDs are as follows:

(12) $$M=\left[\begin{array}{cc} M^{in} & 0\\ 0 & M^{out} \end{array}\right]$$

(13) $$K=\left[\begin{array}{cc} K^{in} & 0\\ 0 & K^{out} \end{array}\right]$$

(14) $$C=\left[\begin{array}{cc} C^{in} & 0\\ 0 & C^{out} \end{array}\right]$$

(15) $$y\left(t\right)=\left[\begin{array}{cc} y^{in}(t); & y^{out}(t) \end{array}\right]$$

(16) $$F_{v}(t)=\left[\begin{array}{cc} F_{v}^{in}(t); & F_{v}^{out}(t) \end{array}\right]$$

in which **M***^in^* and **M***^out^* are the mass matrices in the two horizontal directions, respectively. Similarly, **K***^in^* and **K***^out^* are the stiffness matrices in the two horizontal directions; **C***^in^* and **C***^out^* are the Rayleigh damping matrices in the two horizontal directions; **y***^in^*(*t*) and **y***^out^*(*t*) are the system displacement in the two horizontal directions; $F_{v}^{in}(t)$ and $F_{v}^{out}(t)$ are the damper forces of the VMDs in the two horizontal directions.

The matrix expression of the damper force of the VMD is given by

(17) $$F_{v}\left(t\right)=\Pi K_{0}\Pi^{T}y\left(t\right)+\Pi C_{0}\Pi^{T}\dot{y}\left(t\right)+\Pi P_{1}\left(t\right)+\Pi P_{2}\left(t\right)$$

in which

(18) $$K_{0}=\mathrm{diag}\left[k_{01},k_{02},\ldots ,k_{0n}\right]$$

(19) $$C_{0}=\mathrm{diag}\left[c_{01},c_{02},\ldots ,c_{0n}\right]$$

(20) $$\Pi =\left[\begin{array}{cccc} \cos\theta_{1} & -\cos\theta_{2} & & \\ & \cos\theta_{2} & \ddots & \\ & & \ddots & -\cos\theta_{n}\\ & & & \cos\theta_{n} \end{array}\right]$$

where diag[·] is a diagonal matrix; **Π** is the position matrix of the damper forces of the VMDs.

The damper force of the Maxwell element can be expressed as:

(21) $$\left\{\begin{array}{c} {\dot{P}}_{1}\left(t\right)+U_{1}P_{1}\left(t\right)=K_{1}\Pi^{T}\dot{y}\left(t\right)\\ {\dot{P}}_{2}\left(t\right)+U_{2}P_{2}\left(t\right)=K_{2}\Pi^{T}\dot{y}\left(t\right) \end{array}\right.$$

where

(22) $$\left\{\begin{array}{c} P_{1}\left(t\right)={\left[p_{11}\left(t\right),p_{12}\left(t\right),\;\ldots ,p_{1n}\left(t\right)\right]}^{T}\\ P_{2}\left(t\right)={\left[p_{21}\left(t\right),p_{22}\left(t\right),\;\ldots ,p_{2n}\left(t\right)\right]}^{T} \end{array}\right.$$

(23) $$\left\{\begin{array}{c} K_{1}=\mathrm{diag}\left[k_{11},k_{12},\ldots ,k_{1n}\right]\\ K_{2}=\mathrm{diag}\left[k_{21},k_{22},\ldots ,k_{2n}\right] \end{array}\right.$$

(24) $$\left\{\begin{array}{c} U_{1}=\mathrm{diag}\left[\mu_{11},\mu_{12},\;\ldots ,\mu_{1n}\right]\\ U_{2}=\mathrm{diag}\left[\mu_{21},\mu_{22},\;\ldots ,\mu_{2n}\right] \end{array}\right.$$

## 4. Nonstationary Stochastic Seismic Responses of TTL-VMD System

### 4.1. Nonstationary Stochastic Seismic Excitation

Seismic motion is a nonstationary stochastic process, and its statistical information varies with time. It typically involves two nonstationary processes, intensity non-stationarity, and frequency non-stationarity, which can be defined using Fourier–Stieltjes integrals for nonstationary stochastic processes:

(25) $${\ddot{x}}_{g}(\omega ,t)=\int_{-\infty }^{+\infty }a(\omega ,t)e^{i\omega t}dN(\omega )$$

where ${\ddot{x}}_{g}(\omega ,t)$ is the nonstationary stochastic seismic excitation; $i=\sqrt{-1}$; *a*(*ω,t*) denotes the deterministic modulation function for *t* and *ω* and satisfies *a*(*ω*,*t*) = a*(*−ω*,*t*); “*” denotes the complex conjugate; and *N*(*ω*) is an orthogonal incremental process.

The evolutionary power spectral density (EPSD) function $S_{{\ddot{x}}_{g}}\left(\omega ,t\right)$ of the nonstationary stochastic seismic excitation ${\ddot{x}}_{g}(\omega ,t)$ is typically referred to as

(26) $$S_{{\ddot{x}}_{g}}(\omega ,t)={\left|a(\omega ,t)\right|}^{2}S_{{\ddot{x}}_{f}}(\omega )$$

where $S_{{\ddot{x}}_{f}}(\omega )$ denotes the PSD function of a stationary process.

In most engineering calculations, the non-uniform modulation function of the nonstationary stochastic process represented by Equation (26) is approximated by a slowly varying uniform modulation function *a*(*t*) to simplify the analysis [23], such that

(27) $$S_{{\ddot{x}}_{g}}(\omega ,t)={\left|a(t)\right|}^{2}S_{{\ddot{x}}_{f}}(\omega )$$

where the uniform modulation function *a*(*t*) is defined as

(28) $$a\left(t\right)=\left\{\begin{array}{cc} {\left(t/t_{1}\right)}^{2} & 0\leq t\leq t_{1}\\ 1 & t_{1}\leq t\leq t_{2}\\ \exp\left(c\left(t-t_{2}\right)\right) & t_{2}\leq t \end{array}\right.$$

The PSD function $S_{{\ddot{x}}_{f}}(\omega )$ is modeled using the Kanai–Tajimi spectrum [36]:

(29) $$S_{{\ddot{x}}_{f}}\left(\omega \right)=S_{0}\frac{4\xi_{g}^{2}\omega_{g}^{2}\omega^{2}+\omega_{g}^{4}}{{\left(\omega_{g}^{2}-\omega^{2}\right)}^{2}+4\xi_{g}^{2}\omega_{g}^{2}\omega^{2}}$$

where *ω_g_* is the ground filter frequency; *ξ_g_* is the ground filter damping ratio; and *S*_0_ is the spectral intensity coefficient.

### 4.2. Nonstationary Stochastic Seismic Responses

To be an efficient and accurate solution for stochastic vibration equations, the PEM can offer an effective approach for seismic response analysis of engineering structures [36]. An analytical framework based on the PEM is developed to analyze the stochastic seismic responses of the TTL-VMD system under nonstationary seismic excitation. The pseudo-excitation of the controlled TTL system $\tilde{F}(\omega ,t)$ is given by

(30) $$\tilde{F}(\omega ,t)=MI\sqrt{S_{{\ddot{x}}_{g}}(\omega ,t)}e^{i\omega t}$$

The pseudo-response $\tilde{y}(\omega ,t)$ of the controlled TTL system is given by

(31) $$M\ddot{\tilde{y}}(\omega ,t)+C\dot{\tilde{y}}(\omega ,t)+K\tilde{y}(\omega ,t)+{\tilde{F}}_{v}(\omega ,t)=\tilde{F}(\omega ,t)$$

(32) $${\tilde{F}}_{v}(\omega ,t)=\Pi K_{0}\Pi^{T}\tilde{y}(\omega ,t)+\Pi C_{0}\Pi^{T}\dot{\tilde{y}}(\omega ,t)+\Pi {\tilde{P}}_{1}(\omega ,t)+\Pi {\tilde{P}}_{2}(\omega ,t)$$

where “~” represents the pseudo-operation symbol of the vector.

The pseudo-response $\tilde{y}(\omega ,t)$ can be obtained by performing a time-history response analysis at each discrete frequency point *ω_j_* (*j* = 1, 2, …, *M*); *M* is the number of frequency points with equal intervals. Given the initial responses of the TTL-VMD system, the equation of motion is rewritten in the state space. Thus, Equations (31) and (32) can be transformed into

(33) $$\dot{v}\left(\omega_{j},t\right)=Hv\left(\omega_{j},t\right)+r\left(\omega_{j},t\right)$$

For the original TTL system without control, the system matrices are

(34) $$v\left(\omega_{j},t\right)=\left[\begin{array}{c} \tilde{y}\left(\omega_{j},t\right)\\ \dot{\tilde{y}}\left(\omega_{j},t\right) \end{array}\right];\;r\left(\omega_{j},t\right)=\left[\begin{array}{c} 0\\ M^{-1}\tilde{F}\left(\omega_{j},t\right) \end{array}\right]$$

(35) $$H=\left[\begin{array}{cc} 0 & I\\ -M^{-1}K & -M^{-1}C \end{array}\right]$$

For the TTL system controlled by VMDs, the system matrices are

(36) $$v\left(\omega_{j},t\right)=\left[\begin{array}{c} \tilde{y}\left(\omega_{j},t\right)\\ \dot{\tilde{y}}\left(\omega_{j},t\right)\\ {\tilde{P}}_{1}\left(\omega_{j},t\right)\\ {\tilde{P}}_{2}\left(\omega_{j},t\right) \end{array}\right];\;r\left(\omega_{j},t\right)=\left[\begin{array}{c} 0\\ M^{-1}\tilde{F}\left(\omega_{j},t\right)\\ 0\\ 0 \end{array}\right]$$

(37) $$H=\left[\begin{array}{cccc} 0 & I & 0 & 0\\ -M^{-1}\left(K+\Pi K_{0}\Pi^{T}\right) & -M^{-1}\left(C+\Pi C_{0}\Pi^{T}\right) & -M^{-1}\Pi & -M^{-1}\Pi \\ 0 & K_{1}\Pi^{T} & -U_{1} & 0\\ 0 & K_{2}\Pi^{T} & 0 & -U_{2} \end{array}\right]$$

To further improve the solution accuracy, a numerical integration algorithm, namely, the precise integration method, is employed for the response calculation of the TTL-VMD system [37]. According to the precise integration method, the load variation within each time step **r**(*ω_j_*,*t*) is linear:

(38) $$r\left(\omega_{j},t\right)=r_{0}\left(\omega_{j},t\right)+r_{1}\left(\omega_{j},t\right)\times \left(t-t_{0}\right)$$

where **r**_0_(*ω_j_*,*t*) and **r**_1_(*ω_j_*,*t*) are parameter vectors determined by **r**(*ω_j_*,*t*), respectively.

Based on the precise integration method, the state vector **v**(*ω_j_*,*t_m_*) at time *t_m_* has the following iterative relationship:

(39) $$\begin{array}{c} v\left(\omega_{j},t_{m}\right)=T\left(\tau \right)\left(v\left(\omega_{j},t_{m-1}\right)+H\left(r_{0}\left(\omega_{j},t_{m-1}\right)+H^{-1}r_{1}\left(\omega_{j},t_{m-1}\right)\right)\right)\\ -H\left(r_{0}\left(\omega_{j},t_{m}\right)+H^{-1}r_{1}\left(\omega_{j},t_{m}\right)+r_{1}\left(\omega_{j},t_{m}\right)\tau \right) \end{array}$$

(40) $$\tau =t_{m+1}-t_{m}$$

where **T**(*τ*) is determined by the precise integration method.

The EPSD matrix of the nonstationary stochastic responses of the TTL-VMD system is

(41) $$S_{vv}\left(\omega_{j},t_{m}\right)=v{\left(\omega_{j},t_{m}\right)}^{*}v{\left(\omega_{j},t_{m}\right)}^{T}$$

The response variance of the controlled system is given by

(42) $$\sigma_{v}^{2}\left(t_{m}\right)=2\int_{0}^{+\infty }S_{vv}\left(\omega ,t_{m}\right)d\omega$$

### 4.3. Extreme Responses of Controlled System

The extreme responses of structures are of utmost concern in engineering design and safety assessment. Thus, extreme responses can be employed in the stochastic seismic control of a TTL system to evaluate the efficacy of the VMDs. To derive the extreme responses, the nonstationary stochastic responses of the TTL-VMD system can be replaced by the equivalent stationary stochastic responses over a specific duration *τ*. Assuming *y*(*t*) is any nonstationary response, its equivalent stationary response is represented by $\bar{y}(t)$. Let the extreme value of the equivalent stationary response over the duration *τ* be ${\bar{y}}_{e}$, with a standard deviation of $\sigma_{\bar{y}}$, and define the dimensionless parameter as $\eta ={\bar{y}}_{e}/\sigma_{\bar{y}}$. Based on the assumption that the number of threshold crossings follows a Poisson process, the probability distribution of *η* is given by

(43) $$P\left(\eta \right)=\exp\left[-\kappa \tau \exp\left(-\frac{\eta^{2}}{2}\right)\right]$$

(44) $$\kappa =\frac{1}{\pi }\sqrt{\frac{\lambda_{2}}{\lambda_{0}}};\;\lambda_{0}=2\int_{0}^{+\infty }S_{\bar{y}\bar{y}}\left(\omega \right)d\omega ;\;\lambda_{2}=2\int_{0}^{+\infty }\omega^{2}S_{\bar{y}\bar{y}}\left(\omega \right)d\omega$$

where $\lambda_{0}$ is the 0th-order spectral moment, which reflects the displacement response variance; $\lambda_{2}$ is the 2nd-order spectral moment, which reflects the acceleration response variance; $S_{\bar{y}\bar{y}}\left(\omega \right)$ is the equivalent stationary PSD over the duration *τ*; and *τ* is defined as the duration during which the intensity exceeds 50% of the peak vibration:

(45) $$S_{\bar{y}\bar{y}}\left(\omega \right)=\frac{1}{\tau }\int_{t_{0}/\sqrt{2}}^{t_{0}/\sqrt{2}+\tau }S_{yy}\left(\omega ,t\right)dt$$

where *S_yy_* is any diagonal element of the EPSD matrix **S_vv_**.

The expected value of *η* and the extreme response ${\bar{y}}_{e}$ are approximately determined based on the following probability distribution:

(46) $$E\left(\eta \right)=\sqrt{2\ln\left(\kappa \tau \right)}+\frac{\gamma }{\sqrt{2\ln\left(\kappa \tau \right)}}$$

(47) $${\bar{y}}_{e}=E\left(\eta \right)\sigma_{\bar{y}}=\left(\sqrt{2\ln\left(\kappa \tau \right)}+\frac{\gamma }{\sqrt{2\ln\left(\kappa \tau \right)}}\right)\sigma_{\bar{y}}$$

where *γ* = 0.5772 is the Euler constant.

To assess the efficacy of VMDs under nonstationary excitation, the reduction rate (*R*) of the extreme responses is defined as

(48) $$R=\frac{{\bar{y}}_{e}^{o}-{\bar{y}}_{e}^{c}}{{\bar{y}}_{e}^{o}}$$

where ${\bar{y}}_{e}^{o}$ and ${\bar{y}}_{e}^{c}$ are the extreme responses of the original system and the controlled system, respectively.

## 5. Case Study

### 5.1. Analytical Parameters

A TTL system in Figure 2 is adopted to verify the validity of the proposed approach using VMDs under nonstationary stochastic seismic excitation. The case study involves a transmission tower with a height of 110 m and a span of 832 m. The cross-sectional area of the transmission line is 71.25 mm^2^, and the density is 1.43 kg/m. The first two natural frequencies of the tower in the in-plane direction are 0.57 Hz and 2.29 Hz, while the counterparts in the out-of-plane direction are 0.65 Hz and 2.25 Hz. The damping ratios of the first two frequencies are set as 0.01.

The parameters for the modulation function of the nonstationary stochastic seismic evolutionary spectrum in Equation (27) are determined based on the ground acceleration power spectrum specified by the Chinese code [38], namely, *t*_1_ = 0.8 s, *t*_2_ = 7 s, and *c* = 0.35. The site type of the TTL system is Ⅱ according to the Chinese code. The parameters for the stationary PSD in Equation (29) are as follows: *ω_g_* = 12.57 rad/s, *ξ_g_ =* 0.72, and *S*_0_ = 250.57 cm^2^/s^3^. The mean of the maximum ground acceleration corresponding to this seismic parameter type is 0.4 g (where g is the gravitational acceleration), which is the earthquake intensity of grade 8 specified by the Chinese code. The precise integration method adopts a time step of 0.01 s. To ensure the convergence of the integrals, the trapezoidal rule is adopted in the numerical integration. The TTL system has a relatively low natural frequency, and the power spectrum decays rapidly at high frequencies, ensuring that the contribution from high-frequency components is negligible. Thus, the cutoff frequency is selected as 60 rad/s. The frequency step is selected as 0.1 rad/s to maintain stability and ensure convergence.

### 5.2. Damper Installation Schemes

Overall, eight VMDs are installed in the tower body. Four dampers are located in the in-plane direction and the other four are located in the out-of-plane direction. The parameters of the VMDs under different service temperatures are listed in Table 1. In reality, the six-parameter model of VMDs allows, unlike a single Kelvin model or Maxwell model, a better description of the variation in the storage modulus $G^{\prime }(\omega )$ and the loss modulus $G^{\prime \prime }(\omega )$ for varying values of the excitation frequency. Indeed, a single Kelvin model or Maxwell model can be calibrated to match exactly the experimental values of $G^{\prime }(\omega )$ and $G^{\prime \prime }(\omega )$ corresponding only to a single value of the frequency at a given temperature. The parameters are determined by fitting experimental data using the least squares approximation under different temperature conditions [34,35].

To investigate the control efficacy of VMDs, four damper installation schemes are taken into consideration. For Scheme No. 1, all eight VMDs are installed at the tower bottom. Four VMDs are on the first floor and the other four dampers are on the second floor. For Scheme No. 2, all the VMDs are evenly installed from the first to the fourth floor with two dampers on each floor. For Scheme No. 3, all the VMDs are evenly installed from the third to the sixth floor with two dampers on each floor, as shown in Figure 5a. For Scheme No. 4, all eight VMDs are installed on top of the tower body, namely, four VMDs on the fifth floor and the other four on the sixth floor.

**Figure 6:**
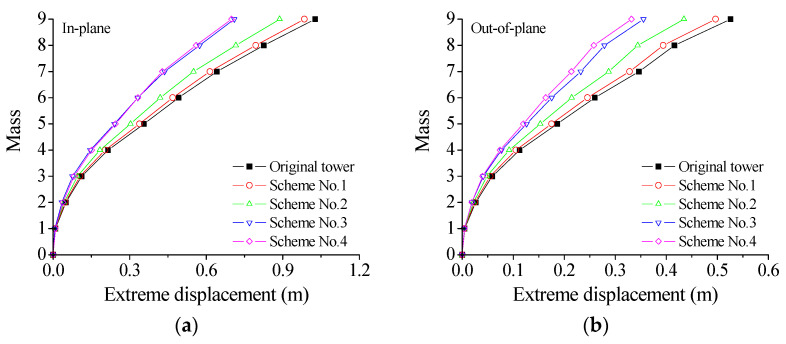

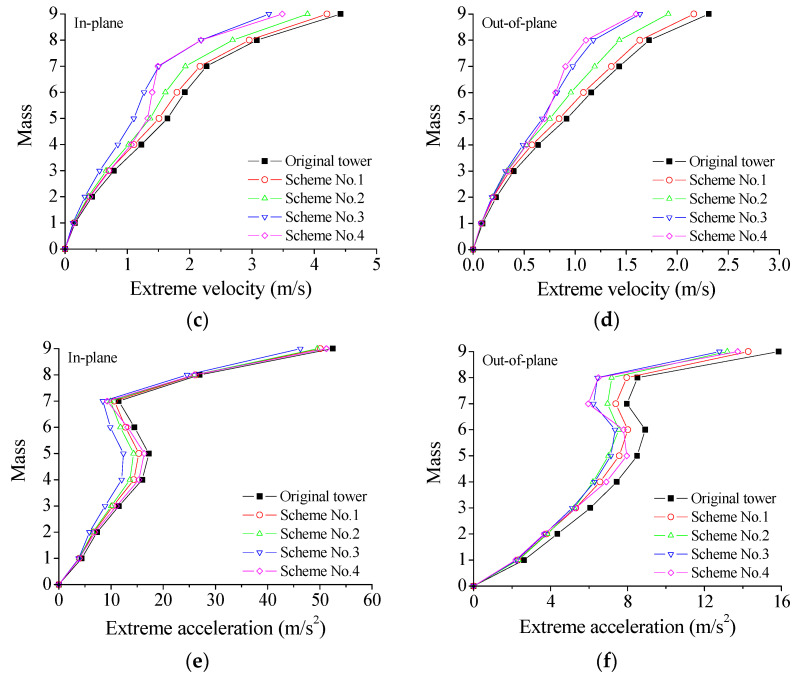
Control efficacy of different schemes: (**a**) extreme displacement; (**b**) extreme displacement; (**c**) extreme velocity; (**d**) extreme velocity; (**e**) extreme acceleration; and (**f**) extreme acceleration.

Figure 6 displays the extreme responses of the tower at an exceeding probability of 0.85 for different control schemes. The labels of the vertical axis in the figure (Mass) represent the number of the concentrated mass layers in the lumped mass model as displayed in Figure 2b, namely, the number of structural dynamic degrees of freedom. The extreme responses of the tower are suppressed substantially with the placement of VMDs. The control effectiveness of the extreme displacement and the extreme velocity are much better than that of the extreme acceleration. In addition, the control effectiveness of the extreme displacement significantly surpasses that of the extreme velocity. The overall efficacy of Schemes No. 3 and 4 is substantially better than that of Schemes No. 1 and 2. The efficacy of Scheme No. 2 is slightly superior to that of Scheme No. 1. Scheme No. 4 exhibits the best control effectiveness in both directions in comparison with the other schemes. Regarding Scheme No. 4, the displacement responses on top of the tower body are much larger than those of the tower bottom. Relatively large floor drifts and damper deformation can be observed. Thus, the energy dissipation and the control efficacy of Scheme No. 4 are satisfactory in comparison with the other three schemes.

The reduction rates of the extreme response of different control schemes are computed and listed in Table 2. The displacement reduction rates of the tower top of Scheme No. 4 are 31.90% and 36.92%, respectively, which are slightly better than the counterparts of Scheme No. 3. Specifically, the reduction rates of the extreme displacement responses in both Scheme No. 3 and 4 exceed 30%, while the reduction rates of extreme velocity responses are also approximately 30%. The overall control performance of Scheme No. 1 and 2 is unsatisfactory because the reduction rates are no more than 20%. The control efficacy of velocity responses is slightly inferior to that of displacement responses, while the reduction rate of acceleration responses is the smallest one. It is noted that structural safety is assessed based on displacement and internal forces. Thus, the reduction rate of displacement responses is the most important index in control performance. In summary, all four damper installation schemes can mitigate the structural seismic responses in both directions. It is noted that the above results were obtained at 21 °C, and similar observations can be made at other service temperatures. A comprehensive evaluation reveals that Scheme No. 4 exhibits a significant advantage in overall control performance, leading to the decision to adopt the damper arrangement of Scheme No. 4 at 21 °C for subsequent parametric studies. The proposed algorithm framework was tested on a computer with the configuration of Intel Core i7-9700 CPU @ 3.00 GHz and 32.0 GB RAM. The calculation times for the uncontrolled and controlled TTL systems are 53 s and 68 s, respectively.

### 5.3. Control Performance

**Figure 7:**
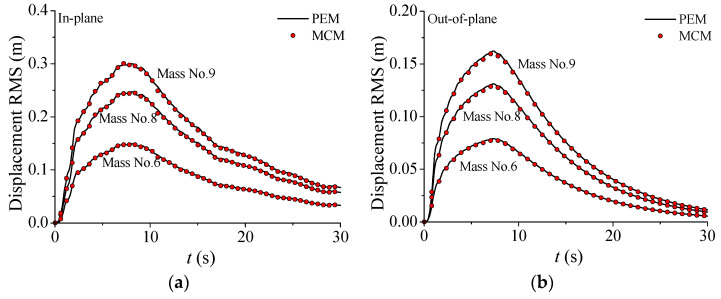

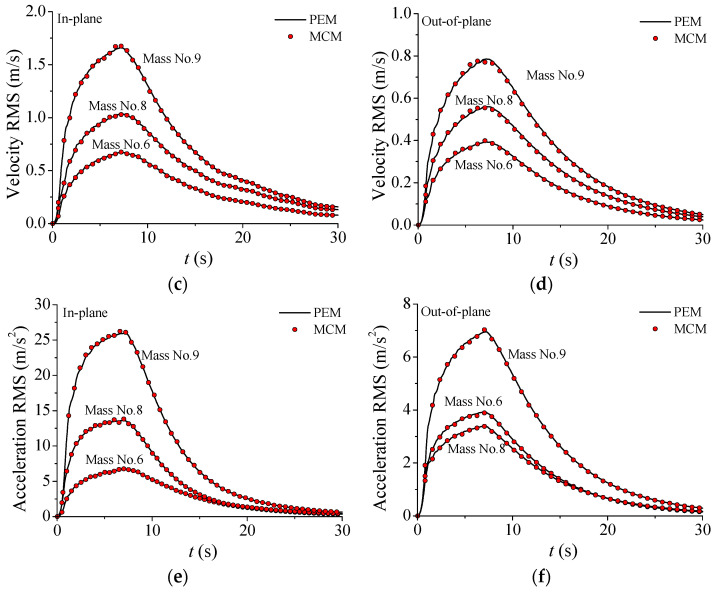
Nonstationary stochastic seismic responses of TTL system: (**a**) displacement RMS; (**b**) displacement RMS; (**c**) velocity RMS; (**d**) velocity RMS; (**e**) acceleration RMS; (**f**) acceleration RMS.

The Monte Carlo method (MCM) was employed to evaluate the accuracy of the proposed nonstationary seismic response analysis framework for the TTL system with VMDs. A numerical simulation was conducted using 5000 samples, followed by statistical analysis of the response RMS at different time instances. The time-varying displacement RMS of the nonstationary responses of the TTL system was computed by PEM in comparison with those based on MCM, as displayed in Figure 7a,b. The observations made with both methods exhibit excellent agreement, thereby validating the accuracy of the computational approach proposed in this study. Similar observations are seen for the velocity and acceleration responses. The response RMS of the TTL system initially increases rapidly and then decreases slowly. In addition, it is seen that the in-plane response RMS is larger than that in the out-of-plane direction for all three types of dynamic responses. This is because the in-plane stiffness of the TTL system is much larger than the out-of-plane stiffness, which is the action of transmission lines. Furthermore, the response RMS of the tower top (Mass No. 9) is much larger than that of the tower body (Mass No. 6), which means that obvious whiplash effects can be observed.

**Figure 8:**
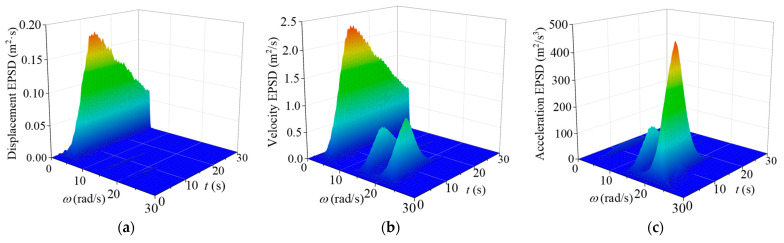

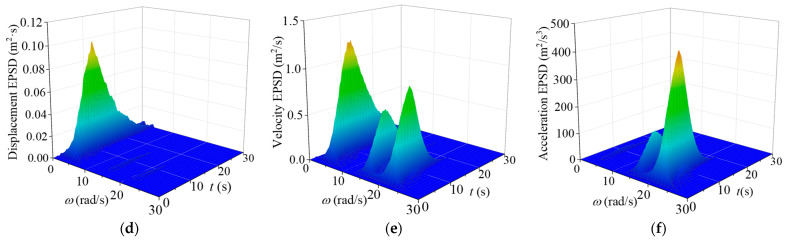
EPSD of structural responses for in-plane vibration: (**a**) displacement EPSD (original tower); (**b**) velocity EPSD (original tower); (**c**) acceleration EPSD (original tower); (**d**) displacement EPSD (with control); (**e**) velocity EPSD (with control); (**f**) acceleration EPSD (with control).

**Figure 9:**
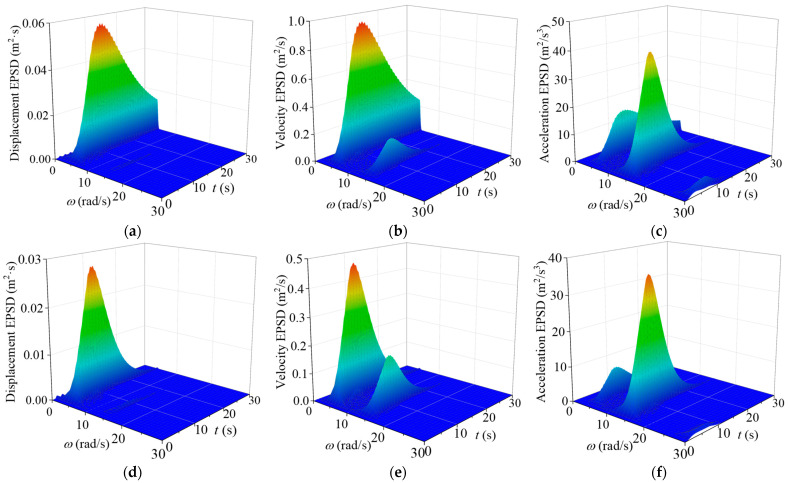
EPSD of structural responses for out-of-plane vibration: (**a**) displacement EPSD (original tower); (**b**) velocity EPSD (original tower); (**c**) acceleration EPSD (original tower); (**d**) displacement EPSD (with control); (**e**) velocity EPSD (with control); (**f**) acceleration EPSD (with control).

The EPSD of the nonstationary seismic responses of the TTL system without and with VMDs for the in-plane vibration are computed and displayed in Figure 8. The comparison of EPSD without and with control indicates that there is no significant change in the structural natural frequencies, suggesting that VMDs have a minimal influence on the inherent vibrational characteristics of the TTL system. It is noted that the spectral amplitude of the controlled tower shows a marked decrease in comparison with that of the original tower. The out-of-plane EPSD presents similar trends, as displayed in Figure 9.

Table 3 lists the extreme values of the EPSD and the reduction rates. Notably, the spectral extreme value of the dynamic responses in both directions shows a remarkable decrease. The data indicate that the reduction rate of the spectrum at the first-order frequency reaches approximately 50%, while the reduction rate at the second-order frequency is significantly lower than that at the first-order frequency. In addition, the out-of-plane reduction rates of the spectra are slightly larger than those for the in-plane vibration, which is similar to the observations of the extreme responses in Table 2. The application of VMDs demonstrates satisfactory control performance in reducing displacement and velocity responses but is less effective in reducing the acceleration response.

## 6. Parametric Study

### 6.1. Effect of Service Temperature

**Figure 10:**
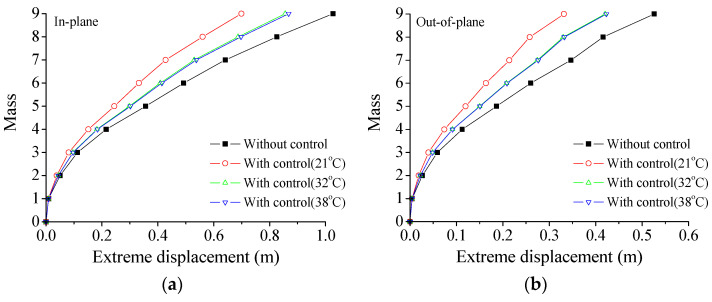

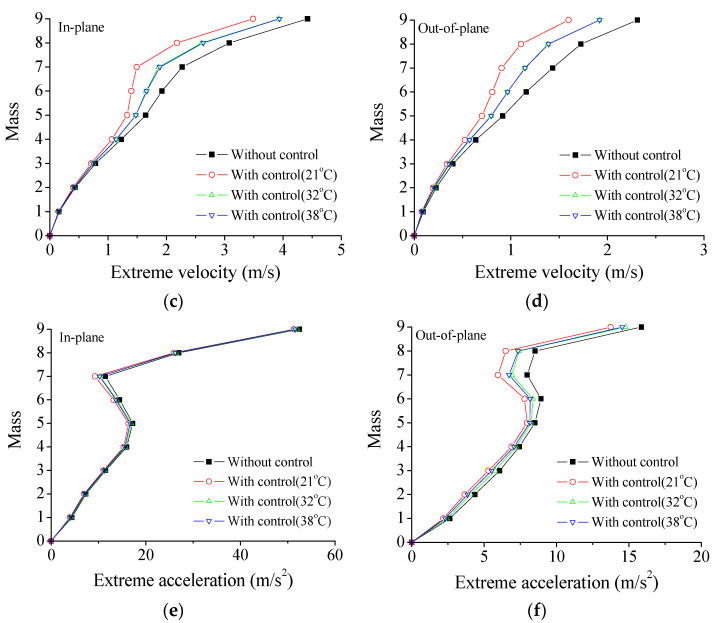
Extreme responses of the tower under different service temperatures: (**a**) extreme displacement; (**b**) extreme displacement; (**c**) extreme velocity; (**d**) extreme velocity; (**e**) extreme acceleration; (**f**) extreme acceleration.

It is reported that the performance of VMDs may vary with the temperatures to some extent [30,31]. Thus, a detailed analysis is conducted on the nonstationary extreme responses of TTL systems under three service temperatures, as shown in Table 1. Figure 10 presents the extreme seismic responses of the tower at an exceeding probability of 0.85 under different service temperatures. The curves indicate that the service temperature significantly affects the VMDs’ performance. The extreme responses gradually reduce with the increasing temperature. Notably, at 21 °C, the extreme seismic responses are smallest compared with those at 32 °C and 38 °C. The effects of temperature on displacement and velocity are much larger than those on acceleration.

The reduction rates of extreme responses at different locations are computed and compared in Table 4 to quantify the influence of the service temperature on the control efficacy. The reduction rates of the extreme displacement at 21 °C are about 32% and 37% for the in-plane and out-of-plane vibration, respectively. The counterparts of the extreme velocity responses at 21 °C are about 21% and 31%, respectively. However, the reduction rates of the extreme responses at 32 °C and 38 °C are substantially lower than that at 21 °C. In addition, the reduction rates of the acceleration responses are much smaller than those of the displacement and velocity responses. Furthermore, the displacement reduction rates surpass those of the velocity and acceleration, confirming that the control performance of the displacement responses is the most effective one. It is noted that the control performance of the VMDs remarkably decreases with the increasing service temperature. This phenomenon can be attributed to the variations in the rheological properties of the viscoelastic material, which result in a decrease in the energy dissipation efficiency of VMDs as the temperature increases. Therefore, it is possible to adopt advanced viscoelastic materials designed to ensure more stable performance of VMDs over a wide thermal range or explore thermal regulation strategies.

### 6.2. Effect of Site Type

The seismic excitations acting on the upper structures are tightly related to the properties of the site type, such as the ground filter frequency, ground filter damping ratio, and the spectral intensity coefficient, as displayed in Equation (29). Therefore, the effects of the site type on the control performance of the TTL system with VMDs are investigated in this section. Listed in Table 5 are the seismic parameters for different site types under earthquake intensity 8. It is noted that the soil becomes softer with increasing site type.

**Figure 11:**
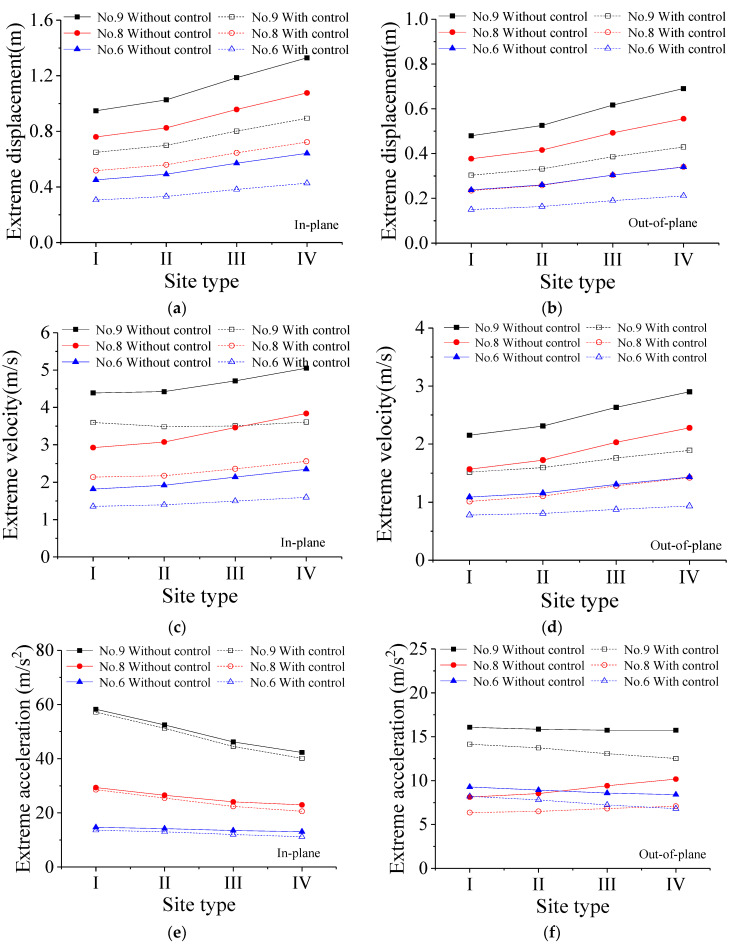
Extreme responses of the tower with different site types: (**a**) extreme displacement; (**b**) extreme displacement; (**c**) extreme velocity; (**d**) extreme velocity; (**e**) extreme acceleration; (**f**) extreme acceleration.

The variations in the extreme responses with the site types are computed and displayed in Figure 11. Data analysis reveals that as soil conditions become softer, both extreme displacement and velocity exhibit a significant increasing trend, while extreme acceleration shows a decreasing trend. This indicates that the site type has a notable influence on the stochastic dynamic responses. It is observed that the VMDs exhibit satisfactory control efficacy for all four site types. The reduction rates of extreme responses with different site types are analyzed and listed in Table 6. It is seen that the reduction rate of extreme displacement exceeds 30%. The reduction rate of extreme velocity is comparatively lower than that of extreme displacement, averaging around 25%. The reduction rate of extreme acceleration is much smaller than the counterparts of extreme displacement and velocity. It is seen that the reduction rates of extreme velocity and acceleration present an upward trend with the increase in the site type number. However, with the increase in the site type number, the soil layer softens, and the reduction rate of extreme displacement stabilizes, indicating a stable control performance on displacement responses.

## 7. Conclusions

This paper proposes a comprehensive analytical framework for the nonstationary stochastic responses of TTL systems with VMDs under stochastic seismic excitations. A detailed parametric investigation was carried out to determine the optimal damper installation scheme and examine the effects of the service temperature and site type on stochastic seismic responses. The conclusions drawn from the case studies are as follows:

(1) The nonstationary stochastic responses of the TTL system are consistent with those based on MCM, validating the accuracy of the proposed analytical framework and demonstrating the effectiveness of VMDs in stochastic response reduction of the TTL system. The VMDs exhibit satisfactory control performance in reducing displacement, while the influence on acceleration is relatively minor.
(2) All four damper installation schemes can mitigate the seismic responses of the TTL system. Scheme No. 4 exhibits the best control effectiveness in both directions in comparison with the other schemes.
(3) Temperature significantly influences the control performance of VMDs, with optimal control effects observed at 21 °C. In contrast, higher temperatures result in a relatively lower reduction rate of stochastic dynamic responses.
(4) The site type has a notable influence on the stochastic seismic responses of the TTL system. Under different site conditions, VMD is effective in controlling the extreme responses of transmission towers, with particularly stable control over displacement. As soil conditions become softer, both extreme displacement and velocity exhibit a significant increasing trend, while extreme acceleration shows a decreasing trend.
(5) It is noted that the proposed comprehensive analytical framework is versatile and applicable to the seismic response analysis of other types of TTL systems with VMDs.

## Figures and Tables

**Table 1:** Parameters of the six-parameter model under different temperatures.

*T* (°C)	*k*_0_ (kN/cm)	*k*_1_ (kN/cm)	*k*_2_ (kN/cm)	*c*_0_ (kN·s/cm)	*c*_1_ (kN·s/cm)	*c*_2_ (kN·s/cm)
21	0.36	42.08	6.87	0.37	0.83	2.15
32	0.21	23.54	3.18	0	0.39	0.73
38	0.28	2327.46	3.28	0	0.57	0.18

**Table 2:** Reduction rates of the extreme response of different control schemes.

Direction	Location	Response	Reduction Rate (%)			
			Scheme No. 1	Scheme No. 2	Scheme No. 3	Scheme No. 4
In-plane	Mass No. 6 (top of tower body)	Displacement	4.71	14.71	32.57	32.49
		Velocity	6.55	16.29	34.08	27.25
		Acceleration	11.51	18.47	31.92	8.76
	Mass No. 8 (cross arm)	Displacement	3.86	13.30	30.53	32.17
		Velocity	3.98	12.62	29.00	29.14
		Acceleration	2.75	3.66	8.95	3.85
	Mass No. 9 (Tower top)	Displacement	4.08	13.54	30.85	31.90
		Velocity	4.97	12.02	26.08	21.17
		Acceleration	4.59	5.69	11.89	2.35
Out-of-plane	Mass No. 6 (top of tower body)	Displacement	5.63	17.44	32.56	37.06
		Velocity	6.62	17.27	28.99	30.25
		Acceleration	10.06	15.33	17.56	12.64
	Mass No. 8 (cross arm)	Displacement	5.24	17.26	32.96	37.97
		Velocity	5.30	16.93	31.67	35.89
		Acceleration	6.57	15.72	24.40	23.84
	Mass No. 9 (tower top)	Displacement	5.54	17.33	32.41	36.92
		Velocity	6.32	17.13	29.23	30.85
		Acceleration	9.93	16.81	19.37	13.39

**Table 3:** Extreme values of EPSD and reduction rates.

Direction	Response	Freq. Order	Extreme Value		Reduction Rate (%)
			Original Tower	With Control	
In-plane	Displacement (m^2^·s)	1	0.1833	0.1002	45.33
		2	0.0035	0.0030	13.17
	Velocity (m^2^/s)	1	2.3491	1.2569	46.50
		2	0.7019	0.6199	11.69
	Acceleration (m^2^/s^3^)	1	30.5683	16.8119	45.00
		2	147.1099	130.4146	11.35
Out-of-plane	Displacement (m^2^·s)	1	0.0581	0.0280	51.71
		2	0.0011	0.0010	12.28
	Velocity (m^2^/s)	1	0.9676	0.4815	50.24
		2	0.2074	0.1841	11.24
	Acceleration (m^2^/s^3^)	1	16.2968	8.3393	48.83
		2	40.9186	36.5927	10.57

**Table 4:** Reduction rates of the extreme response at different service temperatures.

Direction	Location	Response	Reduction Rate (%)		
			21 °C	32 °C	38 °C
In-plane	Mass No. 6 (top of tower body)	Displacement	32.49	16.96	15.73
		Velocity	27.25	14.24	13.71
		Acceleration	8.76	4.03	5.24
	Mass No. 8 (cross arm)	Displacement	32.17	17.45	16.19
		Velocity	29.14	17.89	16.85
		Acceleration	3.85	9.72	10.73
	Mass No. 9 (tower top)	Displacement	31.90	16.67	15.44
		Velocity	21.17	11.20	10.93
		Acceleration	2.35	0.87	1.74
Out-of-plane	Mass No. 6 (top of tower body)	Displacement	37.06	20.12	19.58
		Velocity	30.25	16.57	16.68
		Acceleration	12.64	6.22	8.22
	Mass No. 8 (cross arm)	Displacement	37.97	20.81	20.29
		Velocity	35.89	20.03	19.91
		Acceleration	23.84	13.06	15.56
	Mass No. 9 (tower top)	Displacement	36.92	20.08	19.56
		Velocity	30.85	16.95	16.99
		Acceleration	13.39	6.86	8.36

**Table 5:** Parameters of different site types (earthquake intensity 8).

Site Type	*ω_g_* (Rad/s)	*ξ_g_*	*S*_0_ (cm^2^/s^3^)
I	15.71	0.64	215.19
II	12.57	0.72	250.57
III	8.98	0.80	323.85
IV	6.61	0.90	398.29

**Table 6:** Reduction rates of the extreme responses with different site types.

Direction	Location	Response	Reduction Rate (%)			
			I	II	III	IV
In-plane	Mass No. 6 (top of tower body)	Displacement	32.17	32.49	32.98	33.39
		Velocity	25.43	27.25	30.06	32.04
		Acceleration	7.34	8.76	11.16	14.26
	Mass No. 8 (cross arm)	Displacement	31.94	32.17	32.56	32.87
		Velocity	26.83	29.14	31.78	33.23
		Acceleration	2.60	3.85	7.08	10.34
	Mass No. 9 (tower top)	Displacement	31.50	31.90	32.41	32.73
		Velocity	18.02	21.17	25.63	28.56
		Acceleration	1.83	2.35	3.60	5.13
Out-of-plane	Mass No. 6 (top of tower body)	Displacement	36.83	37.06	37.54	37.92
		Velocity	28.45	30.25	32.98	34.76
		Acceleration	11.49	12.64	15.75	18.99
	Mass No. 8 (cross arm)	Displacement	37.82	37.97	38.32	38.65
		Velocity	35.42	35.89	36.77	37.49
		Acceleration	21.72	23.84	27.60	30.39
	Mass No. 9 (tower top)	Displacement	36.72	36.92	37.39	37.81
		Velocity	29.41	30.85	33.18	34.81
		Acceleration	12.00	13.39	16.94	20.46

## Data Availability

The data generated and/or analyzed during this study are not publicly available due to privacy and our ongoing research.

## Abbreviations

**Symbol**	**Description**
*a*(*t*)	Uniform modulation function for *t*
*a*(*ω,t*)	Modulation function for *t* and *ω*
*A, h*	Total shear area/total thickness
*c*_0_, *c*_1_, *c*_2_	Damping coefficients of the six-parameter model
*c*_0*l*_, *c*_1*l*_, *c*_2*l*_	Damping coefficients of the six-parameter model in the *l*th layer
**C**	Damping matrix of the transmission tower-line system
**C**_0_	Damping matrix composed of all the parameters *c*_0_ in the VMD system
**C***^in^*, **C***^out^*	Damping matrix in the in-plane/out-of-plane direction
*d*; $\dot{d}$	Displacement/velocity of the damper
*d_l_*	Relative displacement between the *l*th layer and the (*l* − 1)th layer of the transmission tower
*F_v_*	Damper force
**F** *_v_*	Damper force vector
$F_{v}^{in}(t)$ $,\;F_{v}^{out}(t)$	Damper force vector of the VMDs in the in-plane/out-of-plane direction
$\tilde{F}(\omega ,t)$	Pseudo-excitation vector of the system
${\tilde{F}}_{v}(\omega ,t)$	Pseudo-damper force vector of the system
*g*	Gravitational acceleration
$G^{\prime }(\omega )$ $,\;G^{\prime \prime }(\omega )$	Shear storage/loss modulus
**H**	State matrix in the equation of state
**I**	Identity matrix
*k*_0_, *k*_1_, *k*_2_	Stiffness coefficients of the six-parameter model
*k*_0*l*_, *k*_1*l*_, *k*_2*l*_	Stiffness coefficients of the six-parameter model in the *l*th layer
*K*′, *K*″	Storage/loss stiffness
**K**	Stiffness matrix of the transmission tower-line system
**K**_0_ **, K**_1_ **, K**_2_	Stiffness matrix composed of all the parameters *k*_0_/*k*_1_/*k*_2_ in the VMD system
$K_{l}^{in}$ $,\;K_{l}^{out}$	Stiffness matrix of the transmission line in the in-plane/out-of-plane direction
**K***^in^*, **K***^out^*	Stiffness matrix in the in-plane/out-of-plane direction
*M*	Number of frequency points with equal intervals
**M**	Mass matrix of the transmission tower-line system
$M_{l}^{in}$ $,\;M_{l}^{out}$	Mass matrix of the transmission line in the in-plane/out-of-plane direction
**M***^in^*, **M***^out^*	Mass matrix of the transmission tower-line system in the in-plane/out-of-plane direction
*n*	Damper number in the tower
*N*(*ω*)	Orthogonal incremental process
*p*_1_, *p*_2_	Damper forces of the Maxwell elements
**P_1_**(*t*), **P_2_**(*t*)	Damper force vectors of the Maxwell element
${\tilde{P}}_{1}\left(\omega ,t\right)$ $,\;{\tilde{P}}_{2}\left(\omega ,t\right)$	Pseudo-damper force vectors of the Maxwell element
$r\left(\omega ,t\right)$	Excitation vector in the equation of state
**r**_0_(*ω*,*t*), **r**_1_(*ω*,*t*)	Parameter vectors determined by **r**(*ω*,*t*)
*R*	Reduction rate of the extreme response
*S*_0_	Spectral intensity coefficient
$S_{{\ddot{x}}_{f}}(\omega )$	Power spectral density function of a stationary process
$S_{{\ddot{x}}_{g}}\left(\omega ,t\right)$	Evolutionary power spectral density function of the nonstationary stochastic seismic excitation
$S_{\bar{y}\bar{y}}\left(\omega \right)$	Equivalent stationary power spectral density function
$S_{vv}\left(\omega ,t\right)$	Power spectral density function of the state vector in the equation of state
*T_c_*, *U_c_*	Kinetic/potential energy of the transmission line
*t*	Time variable
**U**_1_, **U**_2_	Matrix composed of the reciprocal *μ*_1_/*μ*_2_ of the relaxation time of the Maxwell elements
$v\left(\omega ,t\right)$	Pseudo-state vector in the equation of state
*W_c_*(*t*)	Virtual work of the transmission line
${\ddot{x}}_{g}(t)$	Nonstationary seismic acceleration excitation
*y*(*t*)	Nonstationary response
*y_l_*	Displacement of the transmission tower-line system in the *l*th layer
$\bar{y}(t)$	Equivalent stationary response
${\bar{y}}_{e}$	Extreme value of the equivalent stationary response
${\bar{y}}_{e}^{o}$ $,\;{\bar{y}}_{e}^{c}$	Extreme responses of the original/controlled system
y(t) $,\;\dot{y}(t)$ $,\;\ddot{y}(t)$	Displacement/velocity/acceleration responses vector
**y***^in^*(*t*), **y***^out^*(*t*)	Displacement in the in-plane/out-of-plane direction
$\tilde{y}(\omega ,t)$ $,\;\dot{\tilde{y}}(\omega ,t)$	Pseudo-displacement/velocity
τ(t) , γ(t)	Shear stress/strain
$\sigma_{v}^{2}\left(t_{m}\right)$	Response variance of the system at time *t*_m_
*ω*	Circular frequency
*ω_g_*	Ground filter frequency
*ξ_g_*	Ground filter damping ratio
*μ*_1_, *μ*_2_	Reciprocal of the relaxation time of the Maxwell elements
*μ*_1_*_l_*, *μ*_1*l*_	Reciprocal of the relaxation time of the Maxwell elements in the *l*th layer
*θ_l_*	Angle between the damper installed in the *l*th layer
**Π**	Position matrix of the damper forces of the VMDs
$\lambda_{0}$ $,\;\lambda_{2}$	0th-order/2nd-order spectral moment
*τ*	Duration during which the intensity exceeds 50% of the peak vibration
*η*	Ratio of extreme value and standard deviation of the equivalent stationary response
diag[·]	Diagonal matrix
P(·)	Probability distribution
**Acronym**	**Description**
EPSD	Evolutionary power spectral density
MCM	Monte Carlo Method
PEM	Pseudo-excitation method
PSD	Power spectral density
RMS	Root mean square
TTL	Transmission tower-line
TTL-VMD	Transmission tower-line and viscoelastic material damper
VMD	Viscoelastic material damper
3D	Three-dimensional
